# Reserves, resilience and dynamic landscapes 20 years later: *This article belongs to* Ambio*'s 50th Anniversary Collection. Theme: Biodiversity Conservation*

**DOI:** 10.1007/s13280-020-01477-8

**Published:** 2021-02-10

**Authors:** Jan Bengtsson, Per Angelstam, Thomas Elmqvist, Urban Emanuelsson, Carl Folke, Margareta Ihse, Fredrik Moberg, Magnus Nyström

**Affiliations:** 1 Department of Ecology, SLU, 750 07 Uppsala, Sweden; 2 School for Forest Management, Faculty of Forest Sciences, Swedish University of Agricultural Sciences, PO Box 43, 73921 Skinnskatteberg, Sweden; 3 Stockholm Resilience Centre, Stockholm University, Kräftriket 2B, 106 91 Stockholm, Sweden; 4 Department of Urban and Rural Development, Swedish Biodiversity Centre, SLU, Box 7012, 750 07 Uppsala, Sweden; 5 The Beijer Institute, Royal Swedish Academy of Sciences, PO Box 50005, 104 05 Stockholm, Sweden; 6 Department of Physical Geography, Stockholm University, 106 91 Stockholm, Sweden

From the 1970s to the 1990s, ecology was dominated by two separate traditions that viewed ecosystems in different ways, and used different concepts and measures in their research. As Lawton (1994) stated, “For almost three decades, ecosystem and population ecology have ploughed their own independent furrows and developed their own paradigms, approaches and questions”. The population/community ecology paradigm was based on evolutionary theory and looked at changes in numbers and diversity, while ecosystems ecology focused on ecosystem processes like flows of energy and nutrients. During these years, ecology in general also changed in its view of nature, from the early deterministic concepts of climax, stability and balance of nature towards an appreciation of the spatial and temporal dynamics of populations as well as ecosystem processes. These new views emphasised that change was ubiquitous in ecosystems, and that populations, communities and ecosystems were not closed local entities, but embedded in landscapes that changed both through natural processes and, increasingly, human activities (e.g. Worster 1994; Ihse 1995). In the 1990s, there was a rapidly increasing interest in merging the two perspectives, using bridging concepts like food webs, ecosystem engineers and ecosystem functioning (e.g. Jones and Lawton 1995; Polis and Winemiller 1996), and from another angle the emerging resilience concepts based on Holling’s work on ecosystem dynamics (Holling 1973; Folke et al. 1996). Using these concepts, ecologists began asking questions like “What do species do in ecosystems?” (Lawton 1994) and “How does biodiversity matter for ecosystem functioning and ecosystem services?” (Bengtsson et al. 1997; see also Loreau et al. 2001). It was an exciting time for a young ecologist, and year-by-year the distance between basic science and applied questions about landscape management or biodiversity conservation decreased in both minds and actual research.

Our 2003 *Ambio* paper (Bengtsson et al. 2003) grew out of this intellectual melting pot. We attempted to use the basic knowledge about the links between species and ecosystem functioning, drawing on spatial dynamics of populations, landscape ecology, resilience thinking and ecosystem dynamics, to address the problem that much conservation of biodiversity was focusing on preserving local, usually fairly small, reserves and national parks. This was especially so in the large parts of the world where ecosystems had already been drastically altered by human activities, especially intensified land use (Ellis et al. 2010). In much of Europe, North America, Latin America and Africa, protected areas were like islands in a sea of production ecosystems managed by humans, but management rarely considered natural disturbance regimes and the dynamics of ecosystems in general, which over longer time periods occur at larger scales than most reserves. We also mentioned climate change as a problem, stating that “the projected global climatic changes make any reliance on internal recolonization (in local reserves) questionable” (p. 389). Our main conclusion was that for biodiversity and ecosystems in and outside protected areas to reorganise after large-scale disturbances, spatial resilience—which we called ecological memory—in reserves and the surrounding landscape was necessary. We suggested that static reserves should be complemented with dynamic reserves that at the landscape level mimicked the patterns and processes maintained by natural disturbance regimes.^1^

Exactly how the main ideas in the paper emerged is a bit unclear. We had worked together previously, and hence knew each other well enough to trust each other and our different perspectives, but not well enough to fall in the trap of a single perspective. The crucial catalyst that got us going was CBM (the Swedish Biodiversity Centre) which had started in 1995. Both Urban Emanuelsson and Thomas Elmqvist had got jobs there, and were very positive to working together with me and Carl Folke. The paper was long in the making, from 1997 to 2002, but the group that we formed was innovative mainly because of our different perspectives on the major issue. We carried with us experience from community, ecosystems and landscape ecology, from unmanaged and managed terrestrial and marine systems, and conservation in theory and practice. We met in monthly workshops, and despite these meetings often taking off in various directions it was so fun that we always looked forward to them. The slogan “Forwards in all directions!” of the eclectic band 3 Mustaphas 3 from the 1990s applied equally well to us. In the diary of Jan Bengtsson, there is a quote from Carl Folke: “We live 60 years on this earth! How exciting! It should be as fun as this!”. Magnus and Fredrik remember that Carl on the way from Uppsala to Stockholm said something like “Guys, what you have experienced today is very unusual. Don’t expect this is common in science”.

The paper went through several revisions, adding and deleting examples and references and changing structure, before we submitted it to a major conservation journal, from which it bounced back immediately. Then Jan used it in a seminar in a conservation biology course, and the students helped getting it into shape for submitting it to *Ambio*, which was more positive although it took some revisions and almost one more year before it was accepted.

Why has this paper attracted more than 800 citations? The yearly number increased to around 50 in 2014–2015 and in 2019 still around ten. The paper lacks data (although each of us brought our own empirical experience). The theories we used were not new within their respective fields. Our guess is that it was the combination of perspectives and the conceptual vision of combining population, communities and ecosystems ecology, and placing it in a landscape and conservation perspective, that is the reason for the article still being cited. It was at the time also quite novel in many areas of the world to put management of reserves and protected areas in the context of natural and anthropogenic disturbances at larger spatial scales, although this had been suggested before, in the context of designing reserves according to “minimum dynamic areas” (Pickett and Thompson 1978), and was also advocated in the CBD work quite early on. Our main novel argument was that in most landscapes (and seascapes) that have been transformed by human activities or experience considerable human pressures—estimated to be around ¾ of the land area (e.g. Ellis et al. 2010)—biodiversity management and planning cannot rely only on reserves, but must also include the world’s production ecosystems. Our article presaged the land-sparing/land-sharing debate, and actually took a stand that included both; reserves are needed but biodiversity management should also include the production ecosystems that are important for ecosystem services. This idea still appears to be controversial in parts of conservation science, as well as within the productivist paradigm of agriculture and forestry (e.g. Kröger and Raitio 2017; Angelstam et al. 2020).

The dynamic nature of ecosystems, also within reserves (to the dismay of some conservation biologists), will become increasingly important under climate change. For species not to go extinct as the climate becomes warmer they will need to migrate across production landscapes. These landscapes need to have enough suitable habitat forming a functional green infrastructure to allow species to persist. The future of biodiversity and ecosystem services (now also called Nature’s Contributions to People; NCP) thus depends on how well society manages and designs both protected areas and production landscapes.

When reflecting on whether society has met the challenges and ideas developed in the* Ambio* paper, we are both optimistic and pessimistic. While the landscape perspective and the integration of resilience theory and biodiversity conservation is now much more reflected in biodiversity policy (see e.g. IPBES 2019), improvements in conservation practice and landscape management have been quite small, similar to the lack of action to mitigate climate change. The term ecological memory and its importance for recovery and reorganisation after disturbances did not take off; perhaps it was too vague and imprecise. The ideas about using dynamic reserves for landscape management seem to have fallen on bare rocks and withered away, and apart from work on green infrastructure the ideas of large-scale landscape management do not fit well with the focus on provisioning ecosystem services and the sectorisation in society, at least in large parts of Europe. Some of our ecosystem management ideas have been used in a new marine national park in Sweden, as well as when re-zoning the Great Barrier Reef Marine Park, but otherwise we feel that much conservation and landscape planning is still made within a static paradigm that separates protected areas and the rest of the landscape, to the detriment of both. But perhaps things are changing, just a bit too slowly to meet the double challenge of the biodiversity and climate crises.^2^ Allen et al. (2016) developed the ideas on spatial resilience (Nyström and Folke 2001) that were central to the *Ambio* paper and applied them to real landscapes, and recently Maxwell et al. (2020) argued that protection of mobile marine species and habitats will need “innovative and dynamic tools”.

For us as scientists, the article in *Ambio* had a very large impact. It really changed our ways of thinking. The dynamic view of ecosystems and landscapes, the interaction between conservation and production/supply of ecosystem services to society, the spatial linkages of important ecological processes across the landscape, and the need for a social-ecological systems perspective have followed many of us since we started writing the paper. Firstly, the article spawned some other papers using ideas that were formulated during our workshops but did not find their way into *Ambio*. Thomas put some of our thoughts down in a now well-cited paper on response diversity (Elmqvist et al. 2003), and went on to develop urban ecology with Carl at the Stockholm Resilience Centre. Secondly, the ideas on spatial resilience and homogenisation of biodiversity and ecological processes in human-dominated landscapes have influenced several of us. Magnus and Jan were involved in a project examining the loss of resilience in intensive production ecosystems and their dependence on spatially and temporally external resources (Rist et al. 2014). This paper argued that intensified production systems are dependent on massive human interventions and resources often obtained from distant areas, to be maintained at a narrow and brittle edge of stability. Magnus took this further in his recent exploration of the anatomy and resilience of the global production ecosystem (Nyström et al. 2019). And inspired by our multiple perspectives going in “all directions”, Per began working with multiple landscapes as case studies of social-ecological systems (e.g. Angelstam et al. 2013).

For Jan Bengtsson, this paper was crucial in providing the ideas for research on biodiversity and ecosystem services in agricultural and forest landscapes, and the stubborn emphasis on considering landscape level processes to explain everything from local species composition to the supply of ecosystem services to society (e.g. Bengtsson 2010). He also realised the need to understand the role of the “common biodiversity”^3^ that provides ecosystem services in production ecosystems. The common diversity cannot be managed without a landscape perspective that includes sharing the productive land with the species that our future relies on, as well establishing protected areas for them and our other companion travellers on this planet.

The paper also spawned a number of interesting ecological studies on how soil organisms respond to and recover from disturbances like forest fires and intensive agricultural practices. These have taken Jan to South Africa, Russia and the 2014 fire in Västmanland, Sweden. So, while the paper as such was devoid of data, it directed empirical studies towards then rather understudied phenomena related to the spatiotemporal dynamics of populations, communities and ecosystems, and the recovery of organisms and ecological processes after small- and large-scale natural and human disturbances.

Jan is getting close to retirement now. Being a scientist has—apart from the administration of course—often been interesting and fun, and he has made many friends around the globe. But, looking back, Jan cannot see any other period in his working life that was as exciting, surprising and hilariously fun as those years when we explored common but unknown ground together at these monthly workshops that left us exhausted but very happy and satisfied. We wish that all young scientists will experience something similar at least a couple of times, now that we leave many of the issues raised in *Ambio* paper for them to solve.

## Footnotes

1 It could be argued that policymakers implementing the Convention on Biodiversity (CBD) were already working along the lines of landscape management that we were advocating. However, our experience on the ground, both then and now, although mainly from Europe, was that landscape-wide biodiversity management was in practice absent in the agriculture and forestry-dominated landscapes where we worked. We acknowledge that conservation policy may have been experienced differently in other areas, but—as reported in IPBES (2019)—apart from an increased area of protection globally, most conservation goals have failed despite 40 years of policymaking.

2 Regarding the state of biodiversity globally, different views and interpretations are possible, but it is clear that the situation is more precarious than ever (IPBES 2019). IPBES reports failures in meeting most Aichi targets and substantial decreases in biodiversity and ecosystem services globally. It is a matter of opinion whether we should be happy for the fact that it could have been even worse, or simply acknowledge that up until now there has not been much success. As climate scientist Kevin Anderson stated, we have experienced 25 years of failure, both as regards climate and biodiversity policies.

3 The phrase “common biodiversity” was invented by Anki Weibull who together with Ove Eriksson were crucial as internal reviewers of the *Ambio* paper. It is the part of the total biodiversity that is common (and hence not red-listed) and contributes to the supply of most ecosystem services.
